# Motor Performance Is not Enhanced by Daytime Naps in Older Adults

**DOI:** 10.3389/fnagi.2016.00125

**Published:** 2016-05-31

**Authors:** Winifried Backhaus, Hanna Braass, Thomas Renné, Christian Gerloff, Friedhelm C. Hummel

**Affiliations:** ^1^Brain Imaging and NeuroStimulation (BINS) Laboratory, Department of Neurology, University Medical Center Hamburg-EppendorfHamburg, Germany; ^2^Institute of Clinical Chemistry and Laboratory Medicine, University Medical Center Hamburg-EppendorfHamburg, Germany; ^3^Clinical Chemistry, Department of Molecular Medicine and Surgery, Karolinska InstitutetStockholm, Sweden; ^4^University Sleep Medicine Center Hamburg, University Medical Center Hamburg-Eppendorf and Agaplesion HospitalHamburg, Germany

**Keywords:** procedural memory, sleep, consolidation, nap, aging, sequence, adaptation

## Abstract

The impact of sleep on motor learning in the aging brain was investigated using an experimental diurnal nap setup. As the brain ages several components of learning as well as motor performance change. In addition, aging is also related to sleep architectural changes. This combination of slowed learning processes and impaired sleep behavior raises the question of whether sleep can enhance learning and specifically performance of procedural tasks in healthy, older adults. Previous research was able to show sleep-dependent consolidation overnight for numerous tasks in young adults. Some of these study findings can also be replicated for older adults. This study aims to clarify whether sleep-dependent consolidation can also be found during shorter periods of diurnal sleep. The impact of midday naps on motor consolidation was analyzed by comparing procedural learning using a sequence and a motor adaptation task, in a crossover fashion in healthy, non-sleep deprived, older adults randomly subjected to wake (45 min), short nap (10–20 min sleep) or long nap (50–70 min sleep) conditions. Older adults exhibited learning gains, these were not found to be sleep-dependent in either task. The results suggest that daytime naps do not have an impact on performance and motor learning in an aging population.

## Introduction

The capacity of older adults to learn and stabilize motor memory traces (consolidation) during sleep is limited (Backhaus et al., 2015; Pan and Rickard, 2015). A number of studies suggest that sleep-dependent consolidation in healthy older adults is apparent either immediately after sleep (Al-Sharman and Siengsukon, 2014; Gudberg et al., 2015) or after a delay (Tucker et al., 2011; Korman et al., 2015; Mantua et al., 2016). Interestingly these results also hold true for older adults after stroke (Siengsukon and Boyd, 2009). To analyze whether sleep-dependent consolidation can be shown in healthy older adults after a sleep period shorter than that of night sleep, we implemented two different tasks, a sequence learning task and a motor adaptation task, in a diurnal nap study setup.

Sequence learning paradigms are amongst the most commonly implemented designs for assessing sleep-dependent consolidation in various populations. Studies including daytime sleep in older adults combined with motor learning are sparse. Fogel et al. (2014) found that the lack of offline consolidation during napping is the result of changes in the cortico-striatal network and sleep-architecture. The latter includes problems initiating and maintaining sleep and the reduction of total sleep time and sleep efficiency (Ohayon et al., 2004). The impact of sleep duration on a 5-item sequence learning task was revisited by Korman et al. (2015). The authors showed that the total sleep time of a day-night-cycle was increased by implementing a daytime napping paradigm. They found similar offline learning—after 22 h—in young non-napping and older napping adults. This strengthens the link between age-related sleep-architecture changes and limited offline learning. In the current study, different nap durations during sequence learning of a more difficult 9-item sequence task are contrasted to show whether sleep-dependent consolidation can be elicited in diurnal settings in more difficult tasks.

The term “motor adaptation” incorporates the learning of new movements on top of previously known automated movement patterns, leading to a change in learned movement patterns. One example of a motor adaptation tasks are joystick tracking tasks, as previously studied in young adults (Doyon et al., 2009; Backhaus et al., 2016). No sleep-dependent gains could be shown after a daytime nap in younger adults. Similarly no nap-dependent improvements could be shown in young adults for an adaptive whole body movement task (a reverse bicycle steering task; Hoedlmoser et al., 2014). On the other hand previous, especially motor adaptation studies implementing whole hand or whole body movements, showed sleep-dependent consolidation in older adults over night (Al-Sharman and Siengsukon, 2014; Mantua et al., 2016). To bridge this gap in research, the present project aimed to evaluate whether whole hand movement performance can also be enhanced during a shorter period of sleep that is short diurnal sleep.

Whether it is possible to reliably elicit beneficial sleep-dependent consolidation in older adults in the above-mentioned tasks by implementing daytime naps remains uncertain. In the present article, we focus on performance of older adults during sequence learning and motor adaptation. We match the findings to data obtained in younger adults (Backhaus et al., 2016). Based on previous results which showed learning improvements during sequence learning in older adults (Howard and Howard, 1989; Spencer et al., 2007) and findings which showed the capability of older adults to adapt to new movements and movement components (Voelcker-Rehage, 2008), we hypothesized behavioral improvements of older adults in both learning paradigms. In addition, based on findings of night-sleep studies (Al-Sharman and Siengsukon, 2014; Gudberg et al., 2015; Mantua et al., 2016), we expected to observe a difference in the effects of fast offline learning between the intervention groups (sleep/wake), with long nappers showing the greatest performance improvements. Lastly, we predicted increasingly diverging performance curves for the three groups (wake, short nap, long nap) over time.

## Materials and Methods

### Participants

Sixty healthy right-handed older adults (range 60–82 years) were initially invited to the study. Fifty-seven provided informed consent and were randomly placed into one of the following groups: short nap (45 min nap opportunity, 10–20 min sleep), long nap (90 nap opportunity, 50–80 min sleep) or a 45 min period of wakefulness between two learning sessions. The randomization employed the sealed-envelop-method. All participants completed both a sequence learning and a motor adaptation task in a crossover fashion. Participants were excluded in the cases of untreated sleep related disorders (Pittsburgh Sleep Quality Index (PSQI) ≥ 12), recent or the regular use of sleep affecting medication, previous history of neurological or psychiatric disorders, drug abuse or night shift work. Participants were instructed to refrain from caffeine or alcohol a day prior to and on the day of the learning sessions. Participants were also instructed to refrain from napping outside the limits of the study protocol. Sleep habits and possible protocol violations were verbally ascertained prior to each learning session. The study was approved by the local ethics committee (“Ethik-Kommission der Ärztekammer Hamburg”, Germany, PV4596) in accordance with the declaration of Helsinki.

### Procedure

**Figure 1:**
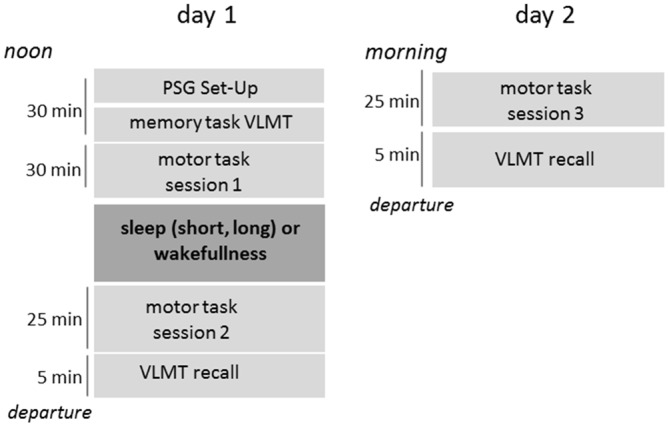
**Timeline of the study design.** PSG, Polysomnography; VLMT, Verbal Learning and Memory Task (declarative).

Sleep and wakefulness was recorded with polysomnography (Alice 3.5, Respironics Inc.) during the midday break and analyzed according to the guidelines of the AASM (Iber et al., 2007). Electrodes were placed at the beginning of session 1, prior to the task introduction (Figure 1). Polysomnographic data was analyzed during the nap to ensure homogeneous sleep duration across groups. This was especially important for the short nap group. In case participants slept past the prescribed nap-length, they were awakened. A final sleep staging was performed after all participants completed the study protocol.

### Motor Tasks

The explicit sequence task (SE) was performed during three sessions, each consisting of seven learning blocks, with a baseline block preceding session 1. A nine-element sequence was displayed on a 60 Hz screen and reproduced using the four fingers of the left hand on a laptop keyboard with covers restricting view to the four relevant buttons. Participants were instructed to repeat the displayed sequence as fast and accurately as possible for the duration of the block (90 s). A dot-cursor highlighted the current position within the sequence. Every block was followed by a 90 s break. Each session included one block with a random sequence which highlighted previous performance improvements. This random block was implemented as the third (first and last session) or fifth block (second session). To ensure equal levels of difficulty, and therefore comparability between the sequences, all employed sequences (learning, baseline and random) had a Kolmogorov complexity index of 1.49. The number of correctly completed sequences was the outcome variable.

To test adaptation skills a target tracking task, motor adaptation task (MA), was implemented. Participants were seated in front of a 70 Hz computer screen, upon which targets (30 × 30 pixel-sized dots) could appear at one out of eight possible predefined locations. These predefined target locations were arranged in circular relationship to the middle of the screen—similar to numbers on a clock—and collected by moving the joystick with the left hand. Joystick movements were in turn projected as a dot-cursor on the screen. A target would disappear when the dot-cursor remained within a 12 pixel radius of the target for at least 100 ms. As soon as the dot-cursor reached its neutral position in the middle of the screen, a new target would appear. Participants were asked to collect as many target dots as possible. Three baseline blocks and a preceding training helped participants adjust to the joystick. After baseline, the joystick movement trajectory was altered by 110° from the 12 o’clock position. Moving the joystick forward then induced cursor movements on the screen in the 110° direction. Each session included one random block with changed joystick deviations (session 1–3: 300°, 60° and 290°). The number of collected dots within the 150 s time frame was the outcome measure.

All learning sessions of both motor tasks were preceded by a reaction time task. Participants had to react as fast as possible to a cue appearing on the display.

### Declarative Learning

A declarative task—the verbal learning and memory test (Helmstädter et al., 2001)—was implemented prior to motor learning (Figure 1). Participants learned lists of 15 words by repeated auditory presentation. Retained knowledge of the list of words was tested at the end of the first and second day.

### Salivary Cortisol

Cortisol is one of the neuromodulators of sleep and was previously found to inhibit declarative memory consolidation during sleep when elevated (Plihal and Born, 1999). Even though this finding could not be replicated during procedural learning (Plihal and Born, 1999; Backhaus and Junghanns, 2006), salivary cortisol was collected prior to the first learning session. It was analyzed with a Roche Cobas Cortisol assay (Roche Diagnostics) and the staff was blinded to group allocation.

### Statistical Analysis

**Figure 2:**
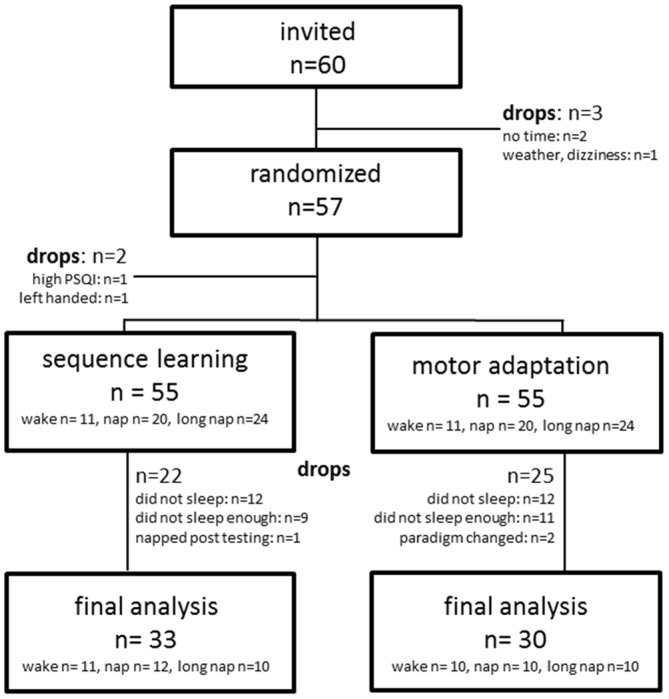
**Recruitment algorithm for sequence learning and motor adaptation**.

Only data of participants that completed the study as randomized were included in the analysis (SE *n* = 33, MA *n* = 30, Figure 2). In addition, a comparison to a previous study with younger adults who performed an identical task was performed. Data analysis was done using SPSS for Windows (IBM, SPSS 22) applying a repeated measures mixed model approach. *Post hoc* testing, after Bonferroni-correction, was pre-defined for the per-protocol analysis. Learning blocks were defined as time points of measurement and serve as continuous independent variable. Sleep duration was defined as fixed effect and participant as a random effect. The model contained all additive main effects for dependent and interaction terms for independent variables. Baseline measures and results from the declarative dataset were collapsed for all participants. Baseline measures were compared with simple *t*-tests adjusting alpha with Bonferroni-correction for multiple comparison. Offline gain was defined as the difference in means between the first and the last learning block encircling either the midday or the nighttime break. Significance level was set to α = 0.05. All data are expressed as mean ± standard deviation unless otherwise indicated.

## Results

### Baseline Measures

To ensure homogeneous intervention groups, participants were screened for sleep quality, depressive symptoms, declarative skills, sleepiness and cortisol level prior to learning (Table 1). At baseline, the groups did not differ in either of these control variables (*p* > 0.1), or in skill level of sequence learning or motor adaptation (*p* > 0.99). Cortisol plasma levels prior to learning were within the normal range with 3.9 ± 2.5 μg/dl (SE) and 3.8 ± 1 μg/dl (MA) for the wake group, 4.3 ± 2.2 μg/dl (SE) and 4.1 ± 1.4 μg/dl (MA) for the short nap group and 3.6 ± 1 μg/dl (SE) and 3.4 ± 1.3 μg/dl (MA) for the long nap group.

**Table 1:** **Baseline data displayed for sequence learning and motor adaptation**.

	Sequence learning			Motor adaptation		
	Wake	Nap	Long nap	Wake	Nap	Long nap
Age in years	73.7 (4.5)	69.9 (6.1)	71.3 (6.0)	74.2 (4.5)	69.3 (6.7)	71.1 (5.5)
Female/Male	9/2	7/5	5/5	9/1	5/5	6/4
Cortisol level (μg/dl)	3.9 (2.5)	4.3 (2.2)	3.6 (1.0)	3.8 (1.0)	4.1 (1.4)	3.4 (1.3)
VMLT 15	46.4 (11.3)	41.5 (7.2)	41.1 (11.3)	46.1 (9.2)	39.0 (14.3)	45.4 (9.1)
BDI	5.8 (4.4)	5.5 (4.8)	4.8 (3.3)	6.2 (4.5)	4.2 (4.3)	5.2 (5.4)
PSQI	4.7 (2.5)	3.5 (2.2)	3.8 (2.0)	5.1 (2.2)	3.0 (2.0)	3.7 (2.2)

*Note: data, derived from all participants included in the analysis is expressed as mean values or absolute counts. () Standard deviation. The groups (wake, nap, long nap) did not differ at baseline*.

### Sleep Parameters

**Figure 3:**
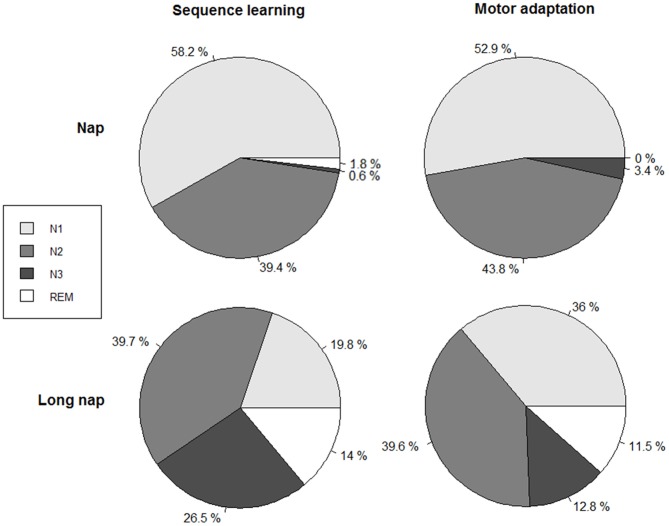
**Relative amount of time spent per sleep stage in relation to the total sleep time, for sequence learning (left column) and motor adaptation (right column).** Total sleep time (TST) nap (explicit sequence task, SE):16.5 ± 2.9 min, nap (motor adaptation task, MA): 14.9 ± 3.0 min, long nap (SE): 59.7 ± 9.1 min, long nap (MA): 60.4 ± 7.1 min.

**Figure 4:**
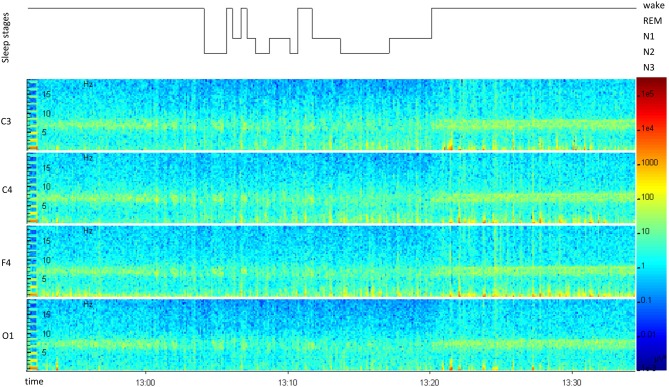
**Representative polysomnographic recording for daytime napping, from “lights off” until “lights on”.** The hypnogram (top) displays the sleep stages over time. A spectral analysis of all recorded scull electrodes (C3, C4, F4, O1) is displayed below.

Participants in the wake group stayed awake for 48.3 ± 3.1 min (SE) or 55.6 ± 18.6 min (MA), between the first two learning sessions. Nappers slept for 16.5 ± 2.9 min (SE), 14.9 ± 3.0 min (MA) in the short nap group and 59.7 ± 9.1 min (SE), 60.4 ± 7.1 min (MA) in the long nap group (Table 2, Figure 3). Participants remained awake for 13.8 ± 7.0 min (SE) or 13.1 ± 8.4 min (MA) prior to the onset of sleep. This was determined by at least one epoch staged in N2 sleep. Long nappers needed slightly longer to fall asleep with 16.3 ± 10.6 min after SE and 16.9 ± 4.3 min after MA. A typical rest period including a short nap is illustrated in Figure 4. Prior to each training and retesting session, the subjective level of sleepiness of each participant was reviewed by completion of the Stanford Sleepiness Scale (Hoddes et al., 1973). No significant differences in levels of alertness within any group emerged across time points before and after the diurnal break (Friedman Test; SE: *p* > 0.083, MA: *p* > 0.414). In the night prior to learning the participants slept around 7.9 ± 1.1 h (SE: wake 8.0 ± 0.9, nap 8.4 ± 1.6, long nap 7.3 ± 0.9, MA: wake 8.4 ± 1.2, nap: 8.2 ± 1.4, long nap 7.9 ± 1.1).

**Table 2:** **Sleep characteristics**.

	Wake	SOL	Stage N1	Stage N2	Stage N3	REM	TST
*Sequence learning*
Wake	48.3 (3.1)	-	0.0 (0.0)	0.0 (0.0)	0.0 (0.0)	0.0 (0.0)	0.0 (0.0)
Nap	36.0 (5.1)	13.8 (7.0)	9.6 (3.5)	6.5 (4.2)	0.1 (0.4)	0.3 (1.0)	16.5 (2.9)
Long nap	61.8 (19.4)	16.3 (10.6)	11.8 (7.5)	23.7 (11.1)	15.9 (16.0)	8.4 (15.9)	59.7 (9.1)
*Motor adaptation*
Wake	55.6 (18.9)	-	0.0 (0.0)	0.0 (0.0)	0.0 (0.0)	0.0 (0.0)	0.0 (0.0)
Nap	35.6 (6.0)	13.1 (8.4)	7.9 (3.7)	6.5 (3.7)	0.5 (1.6)	0.0 (0.0)	14.9 (3.0)
Long nap	61.3 (12.6)	16.9 (4.3)	21.8 (15.1)	23.9 (13.3)	7.8 (8.3)	7.0 (19.3)	60.4 (7.1)

*Note: minutes spent in different sleep stages including means and standard deviations (). SOL: sleep onset latency, TST: total sleep time*.

### Sequence Learning

**Figure 5:**
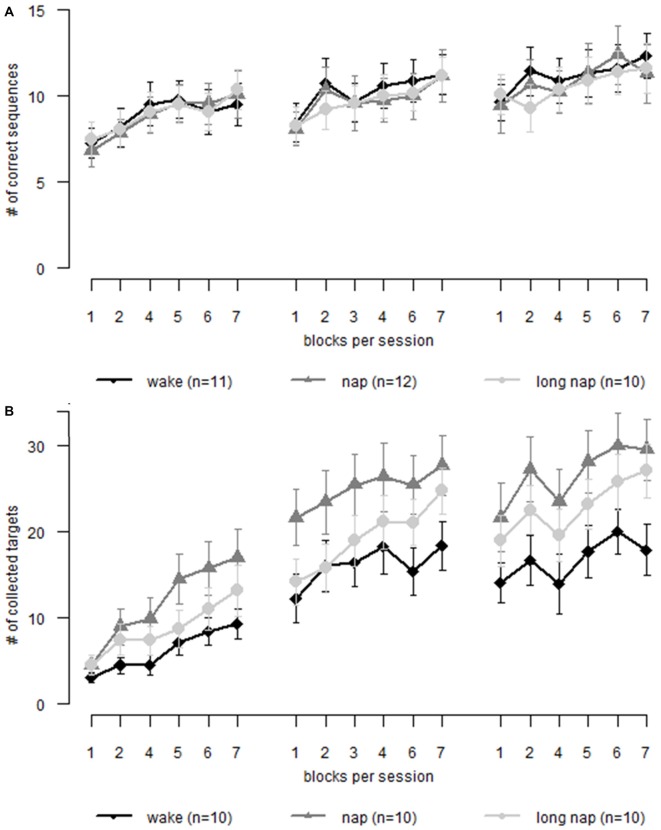
**Motor learning in older adults.** Each graph displays learning during three sessions. Session 1 and 2 are interleaved by a nap or wakefulness, depending on group allocation. Session 2 and 3 is separated by night sleep. **(A)** Sequence learning **(B)** motor adaptation. Error bars represent the standard error of the mean.

While a general learning effect over time (18 blocks of learning throughout three sessions) was found (*F*_(17,48.85)_ = 12.997, *p* < 0.001), napping did not affect motor sequence learning (group * time *F*_(34,48.85)_ = 0.83, *p* = 0.712). We confirmed that actual learning took place by analyzing the random blocks over time; no learning over time was found for the random blocks (*F*_(2,29.78)_ = 1.066, *p* = 0.357). No group showed offline learning, rather offline deterioration. The pre-break skill level was regained after 3–4 blocks of re-learning. The groups did not differ in their offline deterioration during daytime (*p* > 0.99) or nighttime (*p* > 0.99; Figure 5A).

Resulting from inter-individual differences in online learning during session 1, non-significant differences of the pre-break performance levels emerged. The data was corrected in relation to the last block of learning prior to the first diurnal break. A general effect of learning over time remained (*F*_(11,42.4)_ = 6.591, *p* < 0.001), however the interaction with the allocated group was not significant (*F*_(22,42.40)_ = 0.86, *p* = 0.639). There were no significant offline (*p* > 0.701) or online (*p* > 0.294) performance change differences between the groups.

### Motor Adaptation

The analyses showed that participants learned to adapt to the altered joystick movement patterns over time (*F*_(17,41)_ = 61.05, *p* < 0.001). However, there was no significant interaction of group * time (*F*_(34,41)_ = 1.34, *p* = 0.185). No significant offline learning differences between the groups (*F*_(2,27)_ = 0.76, *p* = 0.478), during daytime rest (*p* > 0.287), or during the following period overnight (*p* > 0.99) were apparent. Similarly, the online learning interaction of group * session was not significant (*F*_(4,60.2)_ = 1.158, *p* = 0.338; Figure 5B). The participants were not found to learn differently as a function of the prescribed sleep condition.

To correct for the emerged, non-significant online learning differences (*p* > 0.238) during the first learning session all learning scores were transformed using the individual pre-break performance level. This ensured equal starting performance levels. As in the previous analysis, participants learned to adapt to the joystick over time (*F*_(11,36.8)_ = 9.22, *p* < 0.001) but the groups did not differ at any time point (group * time: *F*_(22,36.8)_ = 1.05, *p* = 0.432). No differences between the groups were found for online (*p* > 0.244) or offline learning (*p* > 0.338).

### Comparing Young and Older Adults Motor Performance—Sequence Learning

**Figure 6:**
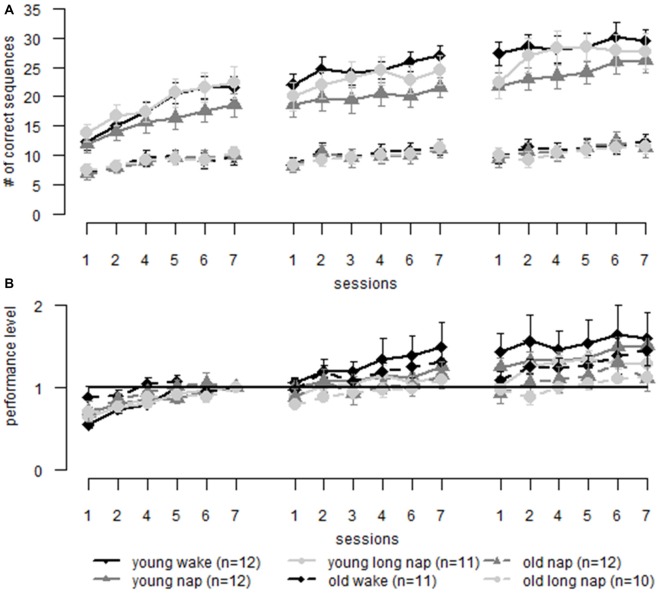
**Sequence learning: comparison of learning in older (dotted lines) and younger adults (solid lines) over three learning sessions including a midday break (first gap) and over-night sleep (second gap). (A)** Raw data comparing young and older adults. **(B)** Learning data is normalized to the same pre-sleep level of learning (last block of the first session) in both young and old adults. Error bars represent the standard error of the mean.

We compared motor performance of younger and older adults during sequence learning. Young adults (Backhaus et al., 2016) performed on a higher level (Figure 6A; group *F*_(1,65.9)_ = 84.05, *p* < 0.001). However, the learning gain was similar in both age groups (Figure 6B; group *F*_(1,59.3)_ = 1.25, *p* = 0.268). *Post hoc* testing showed that when data was adjusted to the last block prior to the midday break, as was also done in previous analyses above, older adults gained significantly less than younger adults during the last session of learning (*p* < 0.034; with the exception of block 6: *p* = 0.081). This suggests age-related deterioration of learning improvements during sequence learning. To answer the question of where these changes arose, offline and online learning changes of non-adjusted data were separated. Similar online performance improvements were found for both age groups during the second session. However, online learning differed significantly between the age groups during the first (young 8.2 ± 4.5, old 2.8 ± 2.9, *p* < 0.001) and the third session (young 3.9 ± 4.4, old 2.1 ± 2.2, *p* = 0.035). Also, a main effect of age was found for offline learning (*F*_(1,128.2)_ = 4.08, *p* = 0.045). *Post hoc* testing remained non-significant. When adjusting the data to the performance level at the end of session one, the differences for online gains vanish and a main effect of age for offline learning remains (*F*_(1,131.9)_ = 6.7, *p* = 0.011), the latter being boosted by overnight learning deterioration (young −0.06 ± 0.3, old −0.2 ± 0.24, *p* = 0.047). These results show that the initial learning session is crucial for age-related differences during sequence learning. However, when this initial learning session is artificially removed, the lacking consolidation during a night of sleep seems to be the driving force for age-related performance changes.

### Comparing Young and Older Adults Motor Performance—Motor Adaptation

**Figure 7:**
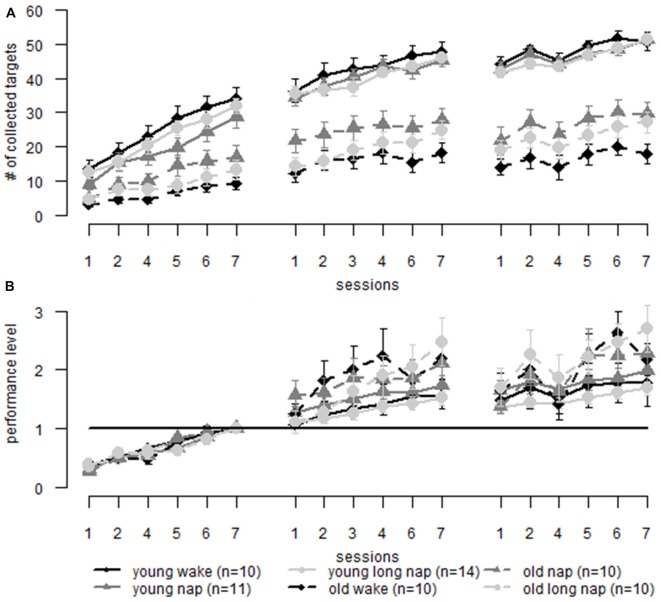
**Motor adaptation: comparison of learning in older (dotted lines) and younger adults (solid lines) over three learning sessions including a midday break (first gap) and over-night sleep (second gap). (A)** Raw data comparing young and older adults.** (B)** Data is normalized to the same pre-sleep level of learning. When the initial learning phase is eliminated the learning curves are similar to older and younger adults. Older adults show greater performance improvements than younger adults. Error bars represent the standard error of the mean.

For the motor adaptation task, the performance of younger (Backhaus et al., 2016) and older adults was compared. The general performance score was affected by age (*F*_(1,63.3)_ = 108.64, *p* < 0.001), with younger adults performing on a higher level than older adults. From the first block on, younger adults collected more targets than older adults (session 1, block 1, young: 11.7 ± 6.86, old: 4.1 ± 3.14, *p* = 0.003; Figure 7A). For better comparability, the last block prior to sleep was used as a new baseline (Figure 7B). Significant differences between the groups emerged starting from session two block two, the second block after the diurnal break, with older adults collecting a relative amount of more dots than younger adults (young: 1.3 ± 0.24, old: 1.6 ± 0.85, *p* = 0.033). These differences between the groups persisted for the remaining second session. There was no significant difference in gain after a night of sleep (young: 1.5 ± 0.55, old: 1.6 ± 0.82, *p* = 0.573). At the end of session three, older adults showed greater gain than younger adults (young: 1.8 ± 0.78, old: 2.3 ± 1.08, *p* = 0.015).

When analyzing non-corrected data, younger adults showed greater online learning than older adults during the first (young 19.8 ± 7.1, old 9.1 ± 7.0, *p* < 0.001) and the second session (young 10.9 ± 6.6, old 7.6 ± 6.3, *p* < 0.044), but not during the final learning session (young 8.4 ± 5.7, old 6.6 ± 5.2, *p* < 0.196). In combination with the previous results, it may be that young adults have reached a performance ceiling in the final session. No differences were found for day- or nighttime offline learning (*p* > 0.150). The results change in the adjusted data where the first learning block was eliminated and the results of the end of this block were used as a new baseline. Older and younger adults differ during the second (young 0.45 ± 0.48, old 0.95 ± 1.1, *p* = 0.014) and third online learning session (young 0.32 ± 0.32, old 0.81 ± 1.1, *p* = 0.014) with older adults performing better than younger adults. Significant differences emerged during offline learning overnight (young −0.12 ± 0.19, old −0.68 ± 1.1, *p* = 0.003), where older adults deteriorate significantly more than younger adults, which is not found during daytime offline learning (young 0.15 ± 0.23, old 0.31 ± 0.66, *p* = 0.229).

### Declarative Learning

Older adults’ knowledge of previously learned words from a 15-item word list deteriorated significantly throughout the experiment (*F*_(2,1.6)_ = 23.11, *p* < 0.001). This change was similar throughout all groups (time point * group *F*_(4,3.24)_ = 0.399, *p* = 0.809).

### Sleep Inertia

Several provisions were made to reduce any confounding effect of sleep inertia. First, participants in the long nap group were given an addition 30 min rest (Tassi and Muzet, 2000) after napping. Second, participants were not awakened. In case their sleep duration would interfere with the group allocation and waking was necessary, then this was not done during slow-wave sleep (Tassi and Muzet, 2000). Finally, a reaction time test was performed prior to each learning session. These results were analyzed using a linear model, including the groups and the reaction times. Significant interactions were not found for either task.

## Discussion

This study, performed to bridge the gap between sleep-dependent consolidation and age-related decline, suggests that short daytime naps do not have an impact on offline learning and sleep-dependent consolidation in older adults. These findings were apparent for both the explicit motor sequence and the motor adaptation learning tasks.

The presented findings of the non-restorative effects of naps are in line with previous research, which was mainly based on sleep-dependent consolidation in older adults during night sleep. Numerous studies could not show a sleep-dependent effect on motor memory consolidation in older adults directly after sleep (Spencer et al., 2007; Siengsukon and Boyd, 2009; Nemeth et al., 2010; Wilson et al., 2012; Pace-Schott and Spencer, 2013; Terpening et al., 2013; Fogel et al., 2014; Gudberg et al., 2015). Age-related changes in sleep-architecture were often found to include the reduction of N2 sleep (Ohayon et al., 2004). Latter leads to the reduction of sleep spindles, a main characteristic feature of N2 sleep (Crowley et al., 2002; Cajochen et al., 2006; Peters et al., 2008). The occurrence of sleep spindles (Milner et al., 2006; Nishida and Walker, 2007; Morin et al., 2008; Tamaki et al., 2008), as well as spindle amplitude (Barakat et al., 2011, 2013), have been previously been linked to procedural learning. The sole change in sleep architecture, also including sleep fragmentation (Ficca et al., 2000), could explain the significantly greater offline performance deterioration found here in older adults, when comparing their performance to younger adults. Nonetheless it should be mentioned that an increase of N2 sleep duration, by affording a daytime nap after learning, was previously hypothesized to enhance sleep-dependent consolidation in older adults after a night of sleep (Korman et al., 2015). Therefore restorative effects of naps in older adults could have also been expected in the current study, but may lack due to differences in the experimental setup.

Sleep-dependent consolidation was previously found in older adults when altering the area of cortical recruitment (whole hand movements (Gudberg et al., 2015), whole body movements (Al-Sharman and Siengsukon, 2014)), when including an additional period of online learning (Tucker et al., 2011) or when the total sleep time was prolonged by including daytime naps (Korman et al., 2015). Based on the latter studies, it was previously hypothesized that a difference between the nap groups may arise after an extended period of consolidation (Tucker et al., 2011; Korman et al., 2015). We were unable to show similar findings in the implemented sequence learning task, opposing results by Tucker et al. (2011) who implemented a 5-element sequence task. Further it has been suggested that increasing the total sleep duration after sequence learning by applying daytime naps would result in similar gains in overnight sleep as in young non-nappers (Korman et al., 2015). We were not able to demonstrate this effect and could also not show differences in offline gains in adults sleeping vs. not sleeping. Perhaps a stratification of the participants concerning their sleep depth at night could have elicited further differentiations between the groups. Overall, our re-test took place the following morning after a full night of sleep, thus about 20 h after the last session, 22 h after the first learning session. The elapsed time is, therefore, similar or even identical to the previously mentioned studies. Discrepancies in the experimental task setup especially concerning task difficulty and the additional learning session after napping may explain some differences in findings. In addition, the interval between learning and retest differs in comparison to previous studies. However, based on research of neurobehavioral tasks (Takahashi and Arito, 2000), these are not expected to have affected any learning outcomes.

Similar to the task presented here, Fogel et al. (2014) implemented a sequence task in a napping paradigm including fMRI measurements. During motor memory consolidation, sequence tasks are thought to rely on cortico-striatal networks and motor adaptation tasks on cortico-cerebellar connections (Doyon et al., 2009). It has been shown that in contrast to young adults, no frontal, parietal or hippocampal activation was present during a post-training nap in older adults—a decrease in activation of the cortico-striatal network was found (Fogel et al., 2014). In addition, older adults have been found to show reduced spindle density during the post-training sleep (Peters et al., 2008; Fogel and Smith, 2011; Fogel et al., 2014). Spindles are associated with motor skill acquisition in young (Peters et al., 2007; Albouy et al., 2008; Morin et al., 2008) but could not be connected to performance gains in older adults (Peters et al., 2008). They were, however, correlated with cerebral activation in older adults (Fogel et al., 2014). The percentage of stage N2 sleep per night was correlated with performance improvements in a mirror-tracing task (Mantua et al., 2016). As our groups (wake/sleep) did not differ from each other during offline learning after the diurnal break we did not perform spindle analyses.

Procedural learning remains important in any age group, for example during rehabilitation after an injury or when dealing with new technologies. It would be very comforting to know that learning could be enhanced by simple napping. Negative emotional memories have previously been found to enhance consolidation during naps in contrast to neutral memories (Nishida et al., 2009) and to enhance procedural skill learning during over-night sleep (Javadi et al., 2011). Perhaps including the amygdala in the consolidation network, for example by implementing more arousing tasks, can lead to desired procedural offline learning during naps when a positive emotional surrounding is provided.

The declarative task was included on the basis that previous studies, especially those employing declarative setups, were able to show sleep-dependent learning. Deterioration, as found here, is a hint that sleep can neither stop decline of trivial knowledge nor enhance non-important declarative content. In addition, it cannot be ruled out that the combination of declarative and motor tasks may hamper the consolidation of each individual task, as was previously seen (Backhaus and Junghanns, 2006; Mednick et al., 2008).

The recuperative values of naps in the aging population has been previously shown (Tamaki et al., 1999; Campbell et al., 2005, 2011; Scullin and Bliwise, 2015). By napping, older adults increase the duration of sleep during 24 h, thus counteracting the effects of sleep fragmentation (Campbell et al., 2005). Thereby, it was found that older adults can align their level of sleeping time (Campbell et al., 2005) and their offline gain (Korman et al., 2015) to young adults without naps. As mentioned previously, we could not confirm the latter finding for healthy, older adults. Nevertheless implementing a napping paradigm is—apart from lacking procedural improvements found in the current study—not a very practical “intervention”. As a result of the problems with the sleep-on-command setup, this study is based on a relatively small sample size per group (*n* ≥ 10), which is nevertheless comparable to previous studies in the field (Siengsukon and Boyd, 2009; Nemeth et al., 2010; Fogel et al., 2014; Gudberg et al., 2015).

Future research should focus not only on the effects of daytime naps in healthy older adults and how these effects can possibly be enhanced, but also on adults with neuropsychiatric diseases, such as stroke as well as on older adults with sleep disorders or sleep deprivation from any cause. In addition, a further exploration of activated networks in these populations—by combining EEG and fMRI measures—is imperative for further research in this field.

## Conclusion

Although midday naps may have a great recuperative value, no positive effects for procedural learning could be demonstrated in the present setup. Based on similar findings of previous research also showing no sleep-dependent consolidation over night or after a nap, as well as the non-apparent differences between the three groups in the present study, we conclude that not the duration of sleep is vital, but rather the process of consolidation during sleep which seems impaired in older adults. Nonetheless, we are confident that the results of this study will assist in answering remaining questions on how motor tasks are readily consolidated during sleep in older adults and how much sleep is needed to elicit offline changes.

## Author Contributions

WB: developing research idea and design, data acquisition and analysis, data interpretation, draft of manuscript. HB: data analysis, critical comments of the manuscript. TR: data analysis, critical comments of the manuscript. CG: interpretation of data, critical comments of the manuscript. FCH: developing research idea and design, data interpretation, critical comments of the manuscript.

## Funding

This work was supported by the Stiftung der Deutschen Wirtschaft providing a Ph.D. scholarship to WB. FCH has been supported by the German Research Foundation (DFG, SFB936/ C4) and by the Federal Ministry of Education and Research (BMBF, TRAINSTIM, Brain Plasticity for Active Aging (01GQ1424B)). WB and FCH hold a research grant from the Werner-Otto-Foundation (9/87). TR acknowledges the German Research Society (SFB877, TP A11 and SFB841, TP B8), and a European Research Council grant (ERC-StG-2012-311575_F-12).

## Conflict of Interest Statement

The authors declare that the research was conducted in the absence of any commercial or financial relationships that could be construed as a potential conflict of interest.

## References

[B1] Albouy G. Sterpenich V. Balteau E. Vandewalle G. Desseilles M. Dang-Vu T.. (2008). Both the hippocampus and striatum are involved in consolidation of motor sequence memory. Neuron 58, 261–272. 10.1016/j.neuron.2008.02.008 18439410

[B2] Al-Sharman A. Siengsukon C. F. (2014). Performance on a functional motor task is enhanced by sleep in middle-aged and older adults. J. Neurol. Phys. Ther. 38, 161–169. 10.1097/NPT.0000000000000048 24866594

[B4] Backhaus W. Braaß H. Renné T. Krüger C. Gerloff C. Hummel F. C. (2016). Daytime sleep has no effect on the time course of motor sequence and visuomotor adaptation learning. Neurobiol. Learn. Mem. 131, 147–154. 10.1016/j.nlm.2016.03.017 27021017

[B3] Backhaus J. Junghanns K. (2006). Daytime naps improve procedural motor memory. Sleep Med. 7, 508–512. 10.1016/j.sleep.2006.04.002 16931152

[B5] Backhaus W. Kempe S. Hummel F. C. (2015). The effect of sleep on motor learning in the aging and stroke population - a systematic review. Restor. Neurol. Neurosci. 34, 153–164. 10.3233/RNN-150521 26835597

[B6] Barakat M. Carrier J. Debas K. Lungu O. Fogel S. Vandewalle G.. (2013). Sleep spindles predict neural and behavioral changes in motor sequence consolidation. Hum. Brain Mapp. 34, 2918–2928. 10.1002/hbm.22116 22674673 PMC6870513

[B7] Barakat M. Doyon J. Debas K. Vandewalle G. Morin A. Poirier G.. (2011). Fast and slow spindle involvement in the consolidation of a new motor sequence. Behav. Brain Res. 217, 117–121. 10.1016/j.bbr.2010.10.019 20974183

[B8] Cajochen C. Münch M. Knoblauch V. Blatter K. Wirz-Justice A. (2006). Age-related changes in the circadian and homeostatic regulation of human sleep. Chronobiol. Int. 23, 461–474. 10.1080/07420520500545813 16687319

[B9] Campbell S. S. Murphy P. J. Stauble T. N. (2005). Effects of a nap on nighttime sleep and waking function in older subjects. J. Am. Geriatr. Soc. 53, 48–53. 10.1111/j.1532-5415.2005.53009.x 15667375

[B10] Campbell S. S. Stanchina M. D. Schlang J. R. Murphy P. J. (2011). Effects of a month-long napping regimen in older individuals. J. Am. Geriatr. Soc. 59, 224–232. 10.1111/j.1532-5415.2010.03264.x 21314644 PMC3074345

[B11] Crowley K. Trinder J. Kim Y. Carrington M. Colrain I. M. (2002). The effects of normal aging on sleep spindle and K-complex production. Clin. Neurophysiol. 113, 1615–1622. 10.1016/s1388-2457(02)00237-7 12350438

[B12] Doyon J. Korman M. Morin A. Dostie V. Hadj Tahar A. Benali H.. (2009). Contribution of night and day sleep vs. simple passage of time to the consolidation of motor sequence and visuomotor adaptation learning. Exp. Brain Res. 195, 15–26. 10.1007/s00221-009-1748-y 19277618 PMC2752878

[B13] Ficca G. Lombardo P. Rossi L. Salzarulo P. (2000). Morning recall of verbal material depends on prior sleep organization. Behav. Brain Res. 112, 159–163. 10.1016/s0166-4328(00)00177-7 10862947

[B14] Fogel S. M. Albouy G. Vien C. Popovicci R. King B. R. Hoge R.. (2014). fMRI and sleep correlates of the age-related impairment in motor memory consolidation. Hum. Brain Mapp. 35, 3625–3645. 10.1002/hbm.22426 24302373 PMC6869653

[B15] Fogel S. M. Smith C. T. (2011). The function of the sleep spindle: a physiological index of intelligence and a mechanism for sleep-dependent memory consolidation. Neurosci. Biobehav. Rev. 35, 1154–1165. 10.1016/j.neubiorev.2010.12.003 21167865

[B16] Gudberg C. Wulff K. Johansen-Berg H. (2015). Sleep-dependent motor memory consolidation in older adults depends on task demands. Neurobiol. Aging 36, 1409–1416. 10.1016/j.neurobiolaging.2014.12.014 25618616 PMC4353561

[B17] Helmstädter C. Lendt M. Lux S. (2001). VLMT Verbaler Lern- und Merkfähigkeitstest (Göttingen: Beltz Test GmbH).

[B18] Hoddes E. Zarcone V. Smythe H. Phillips R. Dement W. C. (1973). Quantification of sleepiness: a new approach. Psychophysiology 10, 431–436. 10.1111/j.1469-8986.1973.tb00801.x 4719486

[B19] Hoedlmoser K. Birklbauer J. Schabus M. Eibenberger P. Rigler S. Mueller E. (2014). The impact of diurnal sleep on the consolidation of a complex gross motor adaptation task. J. Sleep Res. 24, 100–109. 10.1111/jsr.12207 25256866 PMC4491357

[B20] Howard D. V. Howard J. H. Jr. (1989). Age differences in learning serial patterns: direct versus indirect measures. Psychol. Aging 4, 357–364. 10.1037/0882-7974.4.3.357 2803630

[B21] Iber C. Ancoli-Israel S. Chesson A. Quan S. (2007). The AASM Manual for the Scoring of Sleep and Associated Events: Rules, Terminology and Technical Specifications. Westchester, IL: American Academy of Sleep Medicine.

[B22] Javadi A. H. Walsh V. Lewis P. A. (2011). Offline consolidation of procedural skill learning is enhanced by negative emotional content. Exp. Brain Res. 208, 507–517. 10.1007/s00221-010-2497-7 21120459

[B23] Korman M. Dagan Y. Karni A. (2015). Nap it or leave it in the elderly: a nap after practice relaxes age-related limitations in procedural memory consolidation. Neurosci. Lett. 606, 173–176. 10.1016/j.neulet.2015.08.051 26348880

[B24] Mantua J. Baran B. Spencer R. M. C. (2016). Sleep benefits consolidation of visuo-motor adaptation learning in older adults. Exp. Brain Res. 234, 587–595. 10.1007/s00221-015-4490-7 26563162 PMC6398605

[B25] Mednick S. C. Cai D. J. Kanady J. Drummond S. P. A. A. (2008). Comparing the benefits of caffeine, naps and placebo on verbal, motor and perceptual memory. Behav. Brain Res. 193, 79–86. 10.1016/j.bbr.2008.04.028 18554731 PMC2603066

[B26] Milner C. E. Fogel S. M. Cote K. A. (2006). Habitual napping moderates motor performance improvements following a short daytime nap. Biol. Psychol. 73, 141–156. 10.1016/j.biopsycho.2006.01.015 16540232

[B27] Morin A. Doyon J. Dostie V. Barakat M. Hadj Tahar A. Korman M.. (2008). Motor sequence learning increases sleep spindles and fast frequencies in post-training sleep. Sleep 31, 1149–1156. 18714787 PMC2542961

[B28] Nemeth D. Janacsek K. Londe Z. Ullman M. T. Howard D. V. Howard J. H. (2010). Sleep has no critical role in implicit motor sequence learning in young and old adults. Exp. Brain Res. 201, 351–358. 10.3389/conf.fnins.2010.10.00157 19795111

[B30] Nishida M. Pearsall J. Buckner R. L. Walker M. P. (2009). REM sleep, prefrontal theta and the consolidation of human emotional memory. Cereb. Cortex 19, 1158–1166. 10.1093/cercor/bhn155 18832332 PMC2665156

[B29] Nishida M. Walker M. P. (2007). Daytime naps, motor memory consolidation and regionally specific sleep spindles. PLoS One 2:e341. 10.1371/journal.pone.0000341 17406665 PMC1828623

[B31] Ohayon M. M. Carskadon M. A. Guilleminault C. Vitiello M. V. (2004). Meta-analysis of quantitative sleep parameters from childhood to old age in healthy individuals: developing normative sleep values across the human lifespan. Sleep 27, 1255–1273. 15586779 10.1093/sleep/27.7.1255

[B32] Pace-Schott E. F. Spencer R. M. C. (2013). Age-related changes in consolidation of perceptual and muscle-based learning of motor skills. Front. Aging Neurosci. 5:83. 10.3389/fnagi.2013.00083 24348418 PMC3843352

[B33] Pan S. C. Rickard T. C. (2015). Sleep and motor learning: is there room for consolidation? Psychol. Bull. 141, 812–834. 10.1037/bul0000009 25822130

[B35] Peters K. Ray L. Smith V. Smith C. (2008). Changes in the density of stage 2 sleep spindles following motor learning in young and older adults. J. Sleep Res. 17, 23–33. 10.1111/j.1365-2869.2008.00634.x 18275552

[B34] Peters K. R. Smith V. Smith C. T. (2007). Changes in sleep architecture following motor learning depend on initial skill level. J. Cogn. Neurosci. 19, 817–829. 10.1162/jocn.2007.19.5.817 17488206

[B36] Plihal W. Born J. (1999). Memory consolidation in human sleep depends on inhibition of glucocorticoid release. Neuroreport 10, 2741–2747. 10.1097/00001756-199909090-00009 10511433

[B37] Scullin M. K. Bliwise D. L. (2015). Sleep, cognition and normal aging: integrating a half century of multidisciplinary research. Perspect. Psychol. Sci. 10, 97–137. 10.1177/1745691614556680 25620997 PMC4302758

[B38] Siengsukon C. F. Boyd L. A. (2009). Sleep to learn after stroke: implicit and explicit off-line motor learning. Neurosci. Lett. 451, 1–5. 10.1016/j.neulet.2008.12.040 19121365

[B39] Spencer R. M. C. C. Gouw A. M. Ivry R. B. (2007). Age-related decline of sleep-dependent consolidation. Learn. Mem. 14, 480–484. 10.1101/lm.569407 17622650

[B40] Takahashi M. Arito H. (2000). Maintenance of alertness and performance by a brief nap after lunch under prior sleep deficit. Sleep 23, 813–819. 11007448

[B41] Tamaki M. Matsuoka T. Nittono H. Hori T. (2008). Fast sleep spindle (13–15 hz) activity correlates with sleep-dependent improvement in visuomotor performance. Sleep 31, 204–211. 18274267 10.1093/sleep/31.2.204 PMC2225572

[B42] Tamaki M. Shirota A. Tanaka H. Hayashi M. Hori T. (1999). Effects of a daytime nap in the aged. Psychiatry Clin. Neurosci. 53, 273–275. 10.1046/j.1440-1819.1999.00548.x 10459710

[B43] Tassi P. Muzet A. (2000). Sleep inertia. Sleep Med. Rev. 4, 341–353. 10.1053/smrv.2000.0098 12531174

[B44] Terpening Z. Naismith S. Melehan K. Gittins C. Bolitho S. Lewis S. J. G. (2013). The contribution of nocturnal sleep to the consolidation of motor skill learning in healthy ageing and Parkinson’s disease. J. Sleep Res. 22, 398–405. 10.1111/jsr.12028 23398021

[B45] Tucker M. McKinley S. Stickgold R. (2011). Sleep optimizes motor skill in older adults. J. Am. Geriatr. Soc. 59, 603–609. 10.1111/j.1532-5415.2011.03324.x 21410442 PMC4564057

[B46] Voelcker-Rehage C. (2008). Motor-skill learning in older adults—a review of studies on age-related differences. Eur. Rev. Aging Phys. Act. 5, 5–16. 10.1007/s11556-008-0030-9

[B47] Wilson J. K. Baran B. Pace-Schott E. F. Ivry R. B. Spencer R. M. C. (2012). Sleep modulates word-pair learning but not motor sequence learning in healthy older adults. Neurobiol. Aging 33, 991–1000. 10.1016/j.neurobiolaging.2011.06.029 22244090 PMC3307877

